# Workplace bullying and its impact on the quality of healthcare and patient safety

**DOI:** 10.1186/s12960-019-0433-x

**Published:** 2019-11-28

**Authors:** Munirah Al Omar, Mahmoud Salam, Khaled Al-Surimi

**Affiliations:** 1 College of Public Health and Health Informatics, King Saud bin Abdulaziz University for Health Sciences, Riyadh, Saudi Arabia; 2 King Abdullah International Medical Research Center, King Saud bin Abdulaziz University for Health Sciences, Riyadh, Saudi Arabia; 3 Hariri School of Nursing, American University of Beirut, Beirut, Lebanon; 4 Primary Care and Public Health Department, School of Public Health, Imperial College London, London, UK

**Keywords:** Health practitioners, Quality of care, Patient safety, Behavior, Healthcare, Work performance, Work place bullying

## Abstract

**Background:**

Workplace bullying (WPB) is a physical or emotional harm that may negatively affect healthcare services. The aim of this study was to determine to what extent healthcare practitioners in Saudi Arabia worry about WPB and whether it affects the quality of care and patient safety from their perception.

**Methods:**

A cross-sectional study was conducted in 2018. An online survey was distributed among all practitioners at a multi-regional healthcare facility. A previously validated tool was sourced from an integrative literature review by Houck and Colbert. Responses to 15 themes were rated on a 5-point Likert scale, converted to percentage mean scores (PMS) and compared across participants’ characteristics using bivariate and regression analyses.

**Results:**

A total of 1074/1350 (79.5%) completed the questionnaire. The overall median [interquartile range] score of worrying about WPB was 81.7 [35.0]. Participants were mainly worried about the effect of WPB on their stress, work performance, and communication between staff members. A significant negative relationship developed between the quality of care and worrying about WPB, *P* < 0.001. More educated practitioners were 1.7 times more likely to be worried about WPB compared with their counter group, adj.*P* = 0.034. Junior practitioners were 1.6 times more likely to be worried about WPB, adj.*P* = 0.017. The group who has not been trained in handling WPB (1.7 times), and those who had been exposed to WPB (2.2 times) were both more likely to be worried about WPB compared with their counter groups, adj.*P* = 0.026 and adj.*P* < 0.001 respectively.

**Conclusions:**

Most healthcare practitioners worry about WPB, especially its negative impact on the quality of care and patient safety. A greater proportion of practitioners with higher levels of education and their less experienced counterparts were more worried about WPB. Previous exposure to a WPB incident amplifies the practitioners’ worry, but being trained on how to counteract bullying incidents makes them less likely to be worried.

## Key points

- Worries and concerns about workplace bullying (WPB) exist among healthcare workers with varying degrees.
- The healthcare workers holding higher academic credentials and having less work experience were more worried about WPB.
- Previous exposure to WPB significantly raises the level of worry, yet those who have been trained in counteracting bullying incidents were less worried.
- There is a significant association between being worried about WPB and negative rating of the quality/safety of patient care as perceived by healthcare workers.

## Background

Workplace civility is one of the key elements of professionalism in the healthcare industry [1], where an environment of interactive behavior among healthcare practitioners becomes the foundation for a robust hospital performance [2]. Workplace bullying (WPB) is a disruptive behavior that might occur. The World Health Organization (WHO) stated that WPB is an incident during which individuals face a repeated and health-harming mistreatment by perpetrators [3, 4]. It is often characterized by its persistence and long-term duration [5] and caused by psychosocial, cultural, and/or individual factors [6]. WPB includes direct attacks such as hitting, cursing, mocking, or indirect ones such as spreading rumors [7]. One of the major types of bullying is social bullying, i.e., “offensive conduct,” which involves unacceptable jokes related to the gender, public insulting, practical jokes, slander, and exploitation [8].

A multisite study published by the WHO stated that healthcare practitioners, in Brazil 39.5%, in Bulgaria 32.2%, in South Africa 52%, in Thailand 47.7%, in Portugal 27.4%, in Lebanon 40.9%, and in Australia up to 67%, had experienced verbal abuse in a year [9]. Reports showed that the prevalence of WPB among nurses ranged between 27 and 31% [10]. Another study asserted that one in ten family physicians reported an exposure to WPB [11] and that 69.8% of medical residents have been exposed to WPB during their training [12]. In Saudi Arabia, few studies reported that 50–56% of nurses [13, 14], 36.8% of dental interns [15], and 28–84% of medical students or residents reported WPB [16, 17]. Therefore, it is imperative to investigate and analyze the implications of WPB in regions with unique and conservative work cultures such as the Middle East.

While WPB is a common phenomenon worldwide, its implications and tolerability vary according to the cultures, morals, and values of healthcare practitioners embedded in their community and reflected in the healthcare environment. On an individual level, WPB among healthcare workers has been associated with an increase in sickness and/or absenteeism [18]. In retrospect, healthcare practitioners were mostly affected by their stressful work conditions [19]. Likewise, the organizational consequences of WPB can be manifested in a toxic or hostile work environment, which is strongly linked to a compromised quality of care and patient safety. It inhibits teamwork, obstructs communication, disrupts behavior, and increases medical errors by affecting the quality of healthcare organization [20].

Authors of this study believe that determining whether a healthcare practitioner is worried or not about WPB serves a number of purposes. Being worried signifies that either the practitioner has been exposed to WPB, or witnessed an incident encountered by a colleague. Another purpose is that constant worrying over WPB creates a high-risk environment for the employees. It also affects their productivity and level of concentration, which, in turn, increases the likelihood of committing errors and jeopardizing the quality of care and patient safety. Last, worried practitioners might develop fear due to their lack of knowledge about preventing, handling, and reporting a WPB incident. While investigating actual WPB incidents is more reflective of the situation, a survey to determine whether healthcare practitioners are worried about WPB provides some insights into the bullying incidents given that not all WPB incidents are officially reported and documented. Accordingly, there is a pressing need to determine if healthcare practitioners in Saudi Arabia worry about WPB and its effect on the quality of care and patient safety.

## Methods

### Design and setting

Adopting a cross-sectional study, a self-administered survey was distributed among healthcare practitioners at four hospitals in various geographical regions of Saudi Arabia. This large-scale governmental institution can accommodate a total bed capacity exceeding 1000 beds accredited by the Joint Commission International. The healthcare industry in Saudi Arabia comprises local Saudi healthcare workers who serve a relatively religious, tribal, conservative, and culturally oriented community. Female practitioners account for the majority of practitioners working in the Saudi healthcare sector, as reported in two regions of Saudi Arabia (68% and 74.8%) [21, 22]. However, the severe shortage in the workforce has resulted in an influx of expatriates of different races and ethnicities working in the Saudi healthcare industry.

### Study participants

Study participants were all fulltime health practitioners (physicians, nurses, pharmacists, administrative employees, and technicians) and of various career levels, registered under the Saudi Commission for Health Care Specialties. Rules and regulations officially mandate that all healthcare practitioners must communicate in English while on duty. The ethical approval of this study was sought from a governmentally directed Institutional Review Board (SP18/057/R).

In an attempt to maximize the participants’ privacy and comfort, the data collection package comprised an invitation letter, a consent, and an English survey, and made accessible online via SurveyMonkey. A written informed consent was completed by electronically ticking on an “agreement to participate” statement. For convenience, all participants were exposed to the study as the survey was distributed via mass email twice, with a 1-month interval in 2018. Those who participated for the first time were instructed to refrain from participating again. Confidentiality of participants was insured by a disabled tracking of the filed surveys to the participants’ email addresses. One study claimed that 32.8% of healthcare practitioners related WPB to patient safety which indicated that they were worried their patients could be at the receiving end of WPB [23]. A projected sample size of 940 was calculated using the equation (*n* = *t*^2^ × *P*(1 − *P*)/m^2^), where *t* = 1.96 (95% confidence level), *P* = 0.328 [23], and *m* = 0.03 (margin of error 3%).

### Study exposures and outcomes

The survey questionnaire consisted of participants’ socio-demographics and professional characteristics, including gender, age, marital status, level of education, nationality, work duration, job position, and any previous training or exposure to WPB. The WHO defines WPB as a multifaceted form of mistreatment, characterized by the repeated exposure of one person to physical and/or emotional aggression. The participants were instructed on the different forms of bullying: physical, verbal, sexual, or social. Physical bullying causes injuries to an individual’s body or property by beating, kicking, spitting, pinching, pushing, and using rude body language. Verbal bullying involves the use of offensive words through teasing, name-humiliating, and/or unacceptable sexual comments. Relational or social bullying denotes hurting someone’s reputation or relationships via spreading rumors, blatantly ignoring his or her existence, or embarrassing someone in public [3, 7, 24].

Worry has been defined as any negative ideas, images, emotions, or actions that a person has no control of and experiences repetitively. A worried person engages in a proactive cognitive risk analysis of an object, a person, an event, or a situation in attempt to avoid, solve, or anticipate a potential threat [25]. Some believe that worry could be a response to a neutral, mild, or moderate challenge, or even to a non-existing one. From a psychological perspective, a worried person is an anxious individual about a real or imagined issue, such as health, finance, environment, and technology [26].

The level of worry about WPB was assessed using a tool generated by Houck and Colbert, 2017 [23]. Based on an integrative literature review of 36 studies, the two researchers employed the methodology of Whittemore and Knafl to generate a summary of findings or themes related to the quality and safety of patient care all associated with WPB. Houck and Colbert analyzed studies that showed an association between perceived WPB and patient safety and sought evidence of the harmful effects of WPB on the latter. These studies were conducted in North American countries, the UK, and Australia. The main themes identified explored patient falls, errors in treatments or medications, delayed care, adverse event or patient mortality, altered thinking or concentration, silence or inhibits communication, and patient satisfaction or patient complaints [23].

### Data collection

The survey questioned practitioners if they were worried about WPB, the consequences of WPB, and its impact on multiple work aspects. Participants responded to 15 statements (all in negative direction) based on a 5-point Likert scale (strongly disagree = 0, disagree = 1, neutral = 2, agree = 3, and strongly agree = 4). The statements questioned, by and large, whether the practitioners were worried about WPB in terms of work stress, performance, communication, thinking/concentration, delays in delivery of care, medical errors, emotional health, patient complaints, and others. An additional question considered the overall rating of patient safety at their workplace, and responses were rated on a 5-point Likert scale (poor to excellent).

The feasibility and content validity of the questionnaire were tested on a pilot of 16 practitioners. Their subjective feedbacks were analyzed, and their recommended modifications were integrated into the survey to enhance the comprehension of statements. Although this tool is used for the first time in a setting that employs multi-national healthcare practitioners with cultural diversities, its inter-rater reliability test yielded high Cronbach’s alpha of 0.96.

### Statistical analysis

Data entry and analyses were conducted using SPSS v.25 (IBM, NY). Practitioners’ characteristics and their responses to individual statements were presented in frequencies (*n*) and percentages (%). The qualitative responses to the 15 statements were converted to numerical scoring (0 to 4), then summated and presented in percentage mean scores (PMS). The distribution of this PMS was anomalous; outliers were dropped out, and the median [interquartile range (IQR)] was presented. Spearman’s rho was applied to test the relationship between the PMS of worry about WPB and the self-rating of the quality of care/patient safety at workplace (*r*-statistics, *P* value). Mann-Whitney test was used to evaluate the median of worry scores across practitioners’ characteristics, and the *Z*-score was presented (sample size > 30). The worry scores were later categorized into two qualitative outcomes: worried group (PMS > 50) for those who responded agree and strongly agree, and not worried (PMS ≤ 50) for those who responded neutral, disagree, and strongly disagree on the Likert scale. Nurses were grouped with other allied health employees, while pharmacists were grouped with physicians. These two subgroups hold comparable positions in terms of educational levels, scope of practice, and nature of patient care. A binary logistic regression model was constructed to control any potential confounders and to determine the significantly associated factors with the study outcome. The adjusted odds ratio and 95% confidence interval (adj.OR [95% CI]) were presented. Statistical significance was initially set at a *P* value < 0.05, yet corrected using the Holm-Bonferroni method to *P* < 0.025 to counteract for any family-wise error.

## Results

### Sample characteristics

A total of 1074 practitioners completed the questionnaire, including 782 (72.8%) expatriates or non-Saudi employees. Females comprised the majority of the respondents, 922 (85.8%). One third (*n* = 348, 32.4%) were below 30 years old, and half (*n* = 540, 50.3%) were married. A majority of participants (*n* = 704, 65.5%) hold an undergraduate degree, while 207 (19.3%) hold post-graduate degrees. Participants included the following: nurses 519 (48.3%), administrative employees 244 (22.8%), physicians 146 (13.6%), technicians 139 (12.9%), and pharmacists 26 (2.4%). Almost 73% of practitioners had at least 6 years of work experience. Only 118 (11%) of the participants received educational training on workplace bullying, while 684 (63.7%) admitted being exposed to a WPB incident in the past.

### Impact of WPB on work performance and patient safety

Participants were mainly worried about WPB that increased their stress levels. They claimed that WPB negatively affected their work performance and led to communication problems between staff members. Participants’ responses to the statements that assessed various aspects of WPB are enlisted in Table 1. The overall median [IQR] scores of worrying were 81.7 [IQR 35.0]. Most respondents (*n* = 885, 82.4%) can be classified as being worried (PMS > 50).

**Table 1:** Self-reported worrying about WPB

	Strongly Disagree n(%)	Somewhat Disagree n(%)	Neutral n(%)	Somewhat Agree n(%)	Strongly Agree n(%)
Work place bullying increases my stress level.	57(5.3)	34(3.2)	78(7.3)	228(21.2)	677(63.0)
Work place bullying negatively affects my work performance.	67(6.2)	37(3.4)	62(5.8)	293(27.3)	615(57.3)
Work place bullying leads to communication problems among staff.	49(4.6)	43(4.0)	112(10.4)	263(24.5)	607(56.5)
Work place bullying alters my thinking or concentration	59(5.5)	47(4.4)	97(9.0)	258(24.0)	613(57.1)
Work place bullying delays the delivery of care	66(6.1)	45(4.2)	120(11.2)	236(22.0)	607(56.5)
Work place bullying may increases medical errors	58(5.4)	48(4.5)	139(12.9)	224(20.9)	605(56.3)
Work place bullying negatively affects my emotional health.	59(5.5)	55(5.1)	107(10.0)	279(26.0)	574(53.4)
Work place bullying increases patient complaints	64(6.0)	60(5.6)	179(16.7)	226(21.0)	545(50.7)
Work place bullying lowers self-confidence.	83(7.7)	49(4.6)	101(9.4)	355(33.1)	486(45.2)
Work place bullying may lead to increased patient falls.	95(8.8)	54(5.0)	157(14.6)	211(19.7)	557(51.9)
Work place bullying negatively affects my physical health.	97(9.0)	78(7.3)	151(14.1)	241(22.4)	507(47.2)
Work place bullying leads to high rates of adverse events or patient mortality.	106(9.9)	53(4.9)	203(18.9)	203(18.9)	509(47.4)
Work place bullying causes dissatisfaction with my job.	108(10.1)	85(7.9)	128(11.9)	290(27.0)	463(43.1)
Work place bullying makes me consider changing my job.	148(13.8)	75(7.0)	147(13.7)	216(20.1)	488(45.4)
Work place bullying makes me want to stay home rather than go to work.	264(24.6)	107(10.0)	174(16.2)	199(18.5)	330(30.7)

n: frequency, %: percentage

### Factors associated with worrying about WPB

Initial bivariate analysis showed that females 83.3 [IQR 32.1] and younger practitioners 90.0 [IQR 25.0] significantly reported higher scores in comparison with their counter groups, *P* < 0.001 each. Single 86.7 [IQR 28.8] and more educated practitioners 90.0 [IQR 30.0] also had significantly higher scores, *P* = 0.001 each. Practitioners with less work experience also were more worried about WPB, *P* < 0.001. Those who claimed to have had a previous exposure to WPB were significantly more worried, *P* < 0.001, Table 2. A significant negative relationship was found between the practitioners’ self-rating of the quality of care and patient safety at the hospital and the level of worry with WPB (*r* = − 0.433, *P* < 0.001). Binary logistic analyses showed that more educated practitioners were 1.7 times more likely to be worried about WPB compared to their counter groups, adj.*P* = 0.034. Respondents with less experience were 1.6 times more likely to be worried about WPB, adj.*P* = 0.017. Those who have not received previous training on WPB (1.7 times) and those who had been exposed to WPB (2.2 times) were both more likely to be worried compared with their counter groups, adj.*P* = 0.026 and adj.*P* < 0.001 respectively, Table 3.

**Table 2:** Worry scores across health practitioners’ and job characteristics

	n(%) 1074(100)	Median[IQR] 81.7[35]
Gender		
Male	152(14.2)	68.3[41.3]
Female	922(85.8)	83.3[32.1]
		Z=-5.224, P<0.001*
Age (years)		
<30	348(32.4)	90.0[25.0]
≥30	726(68.6)	77.5[40.0]
		Z=-3.826, P<0.001*
Marital status		
Single/Separated	534(49.7)	86.7[28.8]
Married	540(50.3)	78.3[41.7]
		Z=-3.452, P=0.001*
Educational status		
Diploma's/Bachelor’s degree	867(80.7)	80.0[38.3]
Master’s/PhD degree	207(19.3)	90.0[30.0]
		Z=-3.188, P=0.001*
Nationality		
Saudi	292(27.2)	76.7[41.7]
Non-Saudi	782(72.8)	83.3[33.3]
		Z=-2.077, P=0.038
Work experience (years)		
≤10	587(54.7)	88.3[28.3]
>10	487(45.3)	75.0[43.3]
		Z=-5.535, P<0.001*
Job position		
Physicians/Pharmacists	172(16.0)	79.2[41.7]
Nurses/Technicians/Administrative	902(84.0)	81.7[33.3]
		Z=-1.467, P=0.142
Previous training on work place bullying		
Yes	118(11.0)	81.7[40.4]
No	956(89.0)	81.6[33.3]
		Z=-1.341, P=0.180
Previously exposed to WPB		
Yes	684(63.7)	86.7[28.3]
No	390(36.3)	71.7[38.3]
		Z=-9.124, P<0.001*

*IQR* interquartile range, *Z* = Mann-Whitney test *Z*-score, *P value: statistically significant at < 0.025

**Table 3:** Binary logistic regression showing factors associated with worrying about work place bullying

	Worry about work place bullying (PMS>50)		
	B(SE)	Adj.*P*-value	Adj.OR[95%CI]
Gender			
Female vs. Male [ref]	0.25(0.23)	0.130	1.4[0.9-2.2]
Age group			
<30 vs. ≥30 [ref]	0.29(0.25)	0.227	1.3[0.8-2.2]
Marital status			
Single vs. Married [ref]	0.25(0.19)	0.175	1.3[0.9-1.9]
Education level			
Higher vs. Lower [ref]	0.51(0.24)	0.034*	1.7[1.1-2.7]
Work experience			
<10 vs. ≥10 [ref]	0.46(0.19)	0.017*	1.6[1.1-2.3]
Job			
Nurses/allied health vs. Physicians/pharmacists [ref]	0.19(0.25)	0.425	1.2[0.8-2.0]
Previous training			
No vs. Yes [ref]	0.54(0.24)	0.026*	1.7[1.1-2.8]
Previous exposure to incident			
Yes vs. No [ref]	0.79(0.17)	<0.001*	2.2[1.6-3.1]

Adj: adjusted; OR: odds ratio; B: Beta coefficient; SE: standard error; P: *P*-value; *: statistically significant at <0.05; CI: confidence interval; %: percentage; [ref]: reference group

## Discussion

A worried practitioner about WPB might have developed a certain level of anxiety or fear based on an actual bullying incident or a potential escalating situation. The exact prevalence of WPB is debatable and often under-reported [27]. The authors believe that victims of WPB might refrain from disclosing such unfortunate incidents for fear of causing a scandal or jeopardizing their career. While it seems rational for any health practitioner to feel worried about WPB, hospital administrators are compelled to investigate the reasons behind such worries. Similar to other cases, HCWs in this study admitted that WPB affected various themes of patient safety and quality of care (stress management, concentration, workload, communication, delivery of care, medical errors) [28]. Accordingly, this requires a large-scale plan to fight WPB by raising awareness among HCWs, enforcing stringent rules and enacting disciplinary actions. The focus of this study was also to caution hospital administrators about high-risk groups and advise such groups to attend education/training programs on handling/reporting WPB. Furthermore, administrators can attempt to alleviate worker’s concerns and enhance their readiness in case of exposure to WPB.

Female and single practitioners in this multi-regional healthcare facility showed the highest levels of worry about WPB. Female engagement in the workforce has increased drastically in Saudi Arabia, and compared with male workers, females are more prone to WPB [29]. For some practitioners, their need for the job outweighs the aftermath of WPB, yet every hospital administrator should strive to guarantee their safety and satisfaction. The authors also believe that young and single employees might lack some of the skills that help avoid, handle, and confront WPB perpetrators. Surprisingly, more educated practitioners had the highest levels of worry about WPB. One of the possible reasons is that this category of practitioners could be more envied by their colleagues of the same career levels or by their management (downward or upward envy). Envy is a predictor of hostility, competition, or aggressive behaviors which are demonstrated in WPB, and associated with counterproductive work behavior as well [30].

The impact of WPB on practitioners and continuity of patient care is dangerous, not only in terms of errors or substandard care, but also on the high turnover rate for both experienced and inexperienced personnel [31, 32]. A group of practitioners in this multi-regional healthcare facility have admitted that they were, to some degree, exposed to WPB. This group clearly expressed higher levels of worry about WPB being a negative influence on various aspects such as patient care, safety, interaction with other practitioners, and work performance. Even though this self-reported incident occurred at a certain point of time, its emotional residue remains [33]. The authors speculate that a survivor of WPB might develop more experience in handling future incidents. However, personal experience is not enough to guarantee a safe work environment especially if patient care is attenuated by WPB [34]. In this facility, participants had a positive history of WPB, yet their worry scores remained high.

Any issue related to the quality and safety of patient care should not be overlooked since its rating implied a significant relationship with WPB; hence, the responsibility of the hospital administrators is amplified. From the standpoint of practitioners, WPB increases stress, impairs performances, affects communication, and alters thinking, all of which would jeopardize the quality of healthcare and its delivery. Previous literature has proven that the two aspects are significantly linked, as WPB affected both team work and staff retention [23], resulting in depressed practitioners and higher frequency of medical errors [35]. Authors speculate that worrying about WPB exists in all healthcare settings—to a degree—therefore, patient safety can be predicted based on an assessment of this worry. In other words, any patient safety survey or assessment, whether being a key performance indicator, an accreditation pre-requisite, or a research questionnaire should incorporate a domain tackling WPB. For instance, the WHO patient safety tool kit failed to provide any assessment of WPB, which is also neglected by the Safety Competencies to enhance patient safety across the health professions issued by the Canadian Patient Safety Institute [36]. On the other hand, the Registered Nurses Association of Ontario published a better practice guideline which provides recommendations and information about bullying tools [37]. Fifteen evidence-based recommendations for HCWs were displayed so that they can recognize, prevent, and manage WPB and focus on patient care. Therefore, patient safety officers and accrediting bodies are advised to be vigilant with the levels of worry over WPB among health practitioners and to integrate it within their assessment tools.

### Implications for policy, practice, and research

The Saudi Commission for Health Specialties (SCHS) is a national entity that governs and regulates healthcare-related practices in Saudi Arabia. SCHS can ensure that policy-makers and hospital administrators are regularly and closely evaluating the behavior and workplace culture at their facilities. Setting effective zero-tolerance and anti-bullying policies in Saudi Arabia would entail giving SCHS a key role in investigating instances of WPB and revoking work permits of proven perpetrators. Senior leadership must also be involved in introducing and maintaining an effective reporting system for anti-bullying. All Saudi health practitioners should be educated and trained on how to recognize and handle bullying behaviors professionally. Literature has provided evidence for many proven and effective approaches including scripting techniques to diffuse bullying encounters like D.E.S.C. (describe, express, suggest, consequences) or role-play simulation to practice confronting a bully in an assertive manner [38]. Future studies should include testing of the theoretical coherence of the model, and the testing of bullying interventions to determine the effect of bullying on workplace environment and patient-related outcomes.

### Recommendations

Hospital administrators are encouraged to engage in frequent conversations with any practitioner who reports being worried about WPB. Open and transparent personal communication could aid in isolating such unfortunate cases, exposing perpetrators and protecting other employees. The battle against WPB necessitates collaborative efforts between hospital administrators and researchers to further investigate other predisposing factors of WPB that are usually under-reported in hospitals. These include assessing the early childhood of bullies, their psychosocial wellbeing, financial stressors, alcohol/substance abuse, emotional integrity and history of traumas.

This study has several limitations, one of which is generalizability. Despite being conducted across five regions in Saudi Arabia, the selected hospitals are affiliated with one institution. It is worth noting that the native languages of most participants are Arabic, Filipino, Malay, Urdu, and French, but the official language of communication on duty in the targeted facility is English. The survey was relatively easy to comprehend during the pilot stage; however, some practitioners might have misinterpreted some statements. Moreover, some operational definitions, including work performance, stress level, and other terms, stated in the survey, might have been unclear to some study participants. These were sourced from the themes reported by an integrative review paper without alteration by the authors.

Despite the confidentiality and anonymity of participants, some healthcare workers might not have answered the questions honestly which might be attributed to the sensitive nature of the topic. Due to the fact that the survey has been electronically mailed to all healthcare practitioners (office service circulation), the chance of selection bias was minimized. However, some practitioners might have been on vacation or could not have accessed the link for certain reasons. A mismatch in the size of subgroups relating to the sex and job title of participants was present, yet this proportional distribution is reflective of the actual workforce composition at any healthcare institution where the number of nurses outgrows that of physicians, followed by pharmacists and administrative employees. It is also possible that some participants may have responded to the survey multiple times undeterred by the instruction to answer only once. Despite the limitations, this study contributes to the literature of WPB in the Middle East in view of the limited number of studies conducted in this region.

## Conclusions

Most healthcare practitioners worry about WPB, especially its negative impact on the quality of care and patient safety. Being worried about WPB signified that healthcare practitioners were true advocates and safety guardians of their patients. WPB not only exerts stress on practitioners on a personal level but also threatens the patient-practitioner relationship. A greater proportion of practitioners with higher levels of education and those with less working experience were more worried about WPB. Previous exposure to a WPB incident amplifies the practitioner’s worry, but being trained on how to counteract bullying incidents makes them less likely to be worried.

## Data Availability

The datasets generated and/or analyzed during the current study are not publicly available due the institutional rules and regulations, but are available from the corresponding author on reasonable request.

## Abbreviations

95%CI: 95% confidence interval
adj.OR: Adjusted odds ratio
IQR: Interquartile range
PMS: Percentages mean scores
WBI: Workplace Bullying Institute
WPB: Workplace bullying
